# Early acquisition efficiency in visual-to-auditory sensory substitution: the role of structured learning

**DOI:** 10.3389/fpsyg.2026.1887888

**Published:** 2026-08-26

**Authors:** Kyeong Deok Moon, Yun Kyung Park, Moo Seop Kim, Chi Yoon Jeong

**Affiliations:** Sensory-Perception AX Research Section, Electronics and Telecommunications Research Institute (ETRI), Daejeon, Republic of Korea

**Keywords:** acquisition efficiency, cross-modal perception, perception science, perceptual decoding, perceptual learning, psychophysics, structured learning, visual-to-auditory sensory substitution

## Abstract

**Introduction:**

Visual-to-auditory sensory substitution converts visual information into structured sound patterns when visual input is unavailable, impaired, or experimentally restricted, thereby supporting access to information otherwise conveyed visually. Its perceptual usability depends jointly on the sensory-conversion scheme that encodes visual information into sound and on the learning conditions under which learners acquire sound-visual mappings through learning and experience. Although prior visual-to-auditory sensory-substitution studies have often evaluated substituted coding schemes or learning approaches primarily through endpoint learnability or recognition accuracy, endpoint performance alone does not characterize the temporal cost and efficiency with which usable perceptual decoding emerges during learning.

**Methods:**

This study examined whether structured learning based on information-processing principles was associated with more efficient early acquisition of sound-visual mappings in a controlled perceptual-learning model with a constant visual-to-auditory mapping framework. Acquisition-phase behavioral records from 63 blindfolded sighted adults who completed a randomized visual-to-auditory sensory-substitution learning experiment were analyzed, with 31 and 32 participants in the structured-learning and control conditions, respectively. The outcomes included correct-trial response time, total learning time, response-time variability, and inverse efficiency score.

**Results:**

Compared with the control condition, structured learning was associated with shorter correct-trial response time, lower total learning time, and lower inverse efficiency score, without a meaningful between-group difference in response-time variability. These patterns remained evident across robustness analyses and after adjustment for baseline pre-test performance. Exploratory schedule-derived analyses suggested that structured learning provided denser repeated exposure and a more staged introduction of sensory mappings.

**Discussion:**

Together, these behavioral-log findings indicate that structured learning was associated with more efficient early acquisition of sound-visual mappings and suggest that acquisition efficiency in sensory-substitution learning may be related to how those mappings are sequenced and repeated during learning.

## 1 Introduction

Sensory substitution (SS) systems provide a behavioral window into how people interpret information through an alternative sensory channel when a primary sensory pathway is unavailable or experimentally restricted (Auvray, 2025; Bach-y-Rita et al., 1969; Bach-y-Rita and Kercel, 2003; Proulx et al., 2014; Sampaio et al., 2001). Visual-to-auditory sensory substitution (V2A-SS), a form of SS, transforms visual properties such as spatial layout, shape, and contrast into structured auditory patterns that learners must acquire as sound-visual mappings through experience (Abboud et al., 2014; Amedi et al., 2007; Auvray et al., 2007; Haigh et al., 2013; Hanneton et al., 2010; Maidenbaum et al., 2014; Meijer, 1992; Real and Araujo, 2019; Striem-Amit et al., 2012a,b). Because V2A-SS enables the observation of how new sensory mappings are behaviorally acquired, it can serve both as an assistive-technology approach and as a behavioral model for studying cross-modal perception and perceptual learning. Although V2A-SS has demonstrated technical feasibility and proof-of-concept recognition performance, learning effort remains a major issue for broader uptake and practical usability (Maidenbaum et al., 2014; Proulx et al., 2014; Kałwak et al., 2018). Recent studies have therefore emphasized learning, transfer, self-training, mapping flexibility, exploration strategy, self-motion, and task context as factors that shape V2A-SS performance (Akar et al., 2025; Auvray, 2025; Bordeau et al., 2025; Buchs et al., 2021; Jicol et al., 2020; Kucinkas et al., 2026; Stiles et al., 2015).

Despite this growing learning-centered perspective, many sensory-substitution studies still evaluate systems or training approaches mainly through recognition accuracy or other outcomes measured at designated assessment points (Bordeau et al., 2025; Buchs et al., 2021; Jicol et al., 2020; Kim et al., 2024; Kucinkas et al., 2026; Pasqualotto and Esenkaya, 2016). Such endpoint measures are essential for determining whether a substituted code can be learned, but they do not fully describe the temporal cost, response speed, or response stability with which usable sound-visual mappings emerge during acquisition. Two learning approaches may yield similar assessment-point performance while differing substantially in the time required for acquisition, the speed of successful perceptual decoding, or the consistency of responding during learning. From a perception-science and psychophysical perspective, these process-sensitive measures are important because they characterize how efficiently observers adapt to a new sensory code during early exposure (Bruyer and Brysbaert, 2011; Dykiert et al., 2020; Liesefeld and Janczyk, 2019; van der Linden, 2006, 2009, 2019; Vandierendonck, 2017). As summarized in Supplementary Table S1, recent V2A-SS studies have examined recognition, localization, spatial updating, mapping flexibility, self-training, and exploration strategy, yet relatively few have characterized acquisition-phase efficiency using process-sensitive behavioral indicators.

A prior report using the same experimental protocol found that a structured-learning package based on information processing learning theory (IPLT) was associated with better recognition performance at designated assessment points (Moon et al., 2025). That report established the assessment-point learnability of the substituted visual-to-auditory code, but it did not address how efficiently learners acquired usable perceptual decoding during the acquisition phase. The present study addresses this distinct question by analyzing trial-level and activity-level learning records rather than formal assessment-point achievement.

To characterize this acquisition process, the present study used process-sensitive indicators of response speed, temporal learning cost, response stability, and integrated speed-accuracy efficiency. Practice-density analyses were treated as exploratory schedule-derived context rather than as confirmatory tests of mechanism.

Against this background, the present study held the visual-to-auditory mapping framework constant and examined whether structured learning was associated with more efficient early acquisition of sound-visual mappings in a controlled perceptual-learning model. Basic shape categories were used as a controlled substrate for examining foundational image-to-sound mapping acquisition. A specific contribution of this study is that it characterizes V2A-SS acquisition-phase efficiency using a joint profile of response speed, temporal learning cost, response-time variability, and integrated speed-accuracy performance, extending prior V2A-SS research beyond endpoint recognition achievement alone. The study is therefore framed as behavioral evidence on early cross-modal perceptual acquisition, not as direct evidence of neural mechanisms, downstream functional application, or real-world assistive benefit.

## 2 Materials and methods

### 2.1 Design and scope

The present study analyzed acquisition-phase behavioral records obtained from a previously conducted randomized visual-to-auditory sensory-substitution learning experiment. The analysis focused on how efficiently participants acquired visual-to-auditory mappings during learning, rather than on endpoint recognition achievement at formal assessment points. Both groups used the same visual-to-auditory sensory-substitution mapping framework and the same overall stimulus set across six sessions over two weeks, including repeated learning and review sessions and three formal assessments. In the structured-learning condition, participants received a learning package based on information processing learning theory (IPLT), organized into staged study and review modules, whereas the control condition used sequential full-set learning stages without comparable staged introduction, cumulative review, or explicit compare-and-contrast scaffolding. The present analysis therefore estimates acquisition-phase performance associated with the implemented learning package as an integrated whole. The analysis used linked participant-, trial-, activity-, and session-level records, including group assignment and baseline pre-test performance, item-level correctness and response intervals, learning-stage activity time, and session metadata defining the acquisition-phase window. These records supported analyses of learning-phase response time, total learning time, response-time variability, integrated speed-accuracy efficiency, and schedule-derived exposure structure.

The 23 shape categories were organized into six auditory-pattern difficulty levels based on the structural complexity of the corresponding auditory patterns, as described in the prior report (Moon et al., 2025). The present acquisition-phase analysis retained the same fixed Mel-Scaled Frequency Mapping (MSFM)-optimized visual-to-auditory conversion framework used in that study, without modifying the encoding algorithm. The conversion algorithm encoded each 8 × 8 pixel image into a one-second auditory signal by scanning the image column by column from left to right and mapping vertical pixel position to frequency (80–7,600 Hz) and pixel brightness to amplitude, with pixel-level signals synthesized into column sounds and concatenated into the final auditory output. Representative examples of the shape categories and the image-to-sound conversion process are provided in Supplementary Figures S1, 2.

Table 1 summarizes the analytic scope of the present acquisition-phase analysis in relation to the prior achievement-focused report using the same experimental protocol (Moon et al., 2025).

**Table 1:** Comparison of the present acquisition-phase analysis and the prior achievement-focused report using the same experimental protocol.

Dimension	Present manuscript	Prior report (Moon et al., 2025)
Primary objective	Characterize acquisition-phase efficiency and temporal learning cost	Evaluate achievement at designated assessment points
Main research question	Is structured learning associated with more efficient early perceptual decoding during learning?	Is structured learning associated with better recognition performance at assessment points?
Data level analyzed	Trial-, activity-, and session-level acquisition records	Formal assessment-point recognition records
Main outcome measures	Correct-trial RT, total learning time, RT variability, IES, schedule-derived practice density	Assessment-point recognition and achievement outcomes
Analytic approach	Trial-level GEE, robustness analyses, participant-level covariate-adjusted models, exploratory practice-density models	Achievement-focused endpoint analysis
Scientific contribution	Provides behavioral-log evidence on early perceptual decoding efficiency and temporal acquisition cost	Demonstrates assessment-point learnability and achievement benefit of structured learning

RT, response time; IES, inverse efficiency score; GEE, generalized estimating equations.

The present acquisition-phase analysis is best understood as complementary to, rather than duplicative of, that prior report; both studies address distinct inferential questions from the same experimental platform, using different data levels, outcome measures, and analytic approaches.

### 2.2 Participants

A total of 63 participants completed the acquisition-phase protocol: 31 in the structured-learning condition and 32 in the control condition. All participants were healthy sighted adults who completed the experiment under blindfolded conditions. The two groups did not differ meaningfully in age, sex distribution, or baseline pre-test performance. The blindfolded sighted adults were used as a controlled sensory-learning testbed to isolate early acquisition dynamics under reduced heterogeneity, minimizing variability related to residual vision, prior compensatory strategies, and previous device-use experience. The sample should therefore be interpreted as a controlled model of early sensory-substitution learning rather than as a direct model of downstream functional application.

### 2.3 Outcome measures

The primary outcome domain was early acquisition efficiency during V2A-SS learning. Correct-trial response time was the principal indicator of perceptual decoding efficiency, serving to index the speed of successful decoding rather than the overall performance irrespective of accuracy. Total learning time captured the cumulative temporal acquisition cost as the total time spent in learning-stage activities across sessions. Response-time variability was evaluated using the within-participant standard deviation of correct-trial log-transformed response time (Dykiert et al., 2020), with lower values reflecting more consistent response timing.

Inverse efficiency score, calculated by dividing participant-level median correct-trial response time by the corresponding learning-phase accuracy, served as an integrated speed-accuracy index (Bruyer and Brysbaert, 2011; Liesefeld and Janczyk, 2019; Vandierendonck, 2017). Learning-phase accuracy was retained as contextual information and as a component of inverse efficiency score rather than as the main inferential endpoint. Repetition count, cumulative item coverage, recurrence lag, and practice density were the schedule-derived descriptors used to provide exploratory context for the acquisition-efficiency findings.

### 2.4 Time-based outcome handling

Response time was defined as the elapsed time from audio playback completion to answer submission, capturing the post-playback decision-and-response interval without including image-to-audio conversion time or playback duration. Because all task images were converted to audio in advance, system-side conversion latency was excluded from the analyzed response-time measure and treated as contextual technical information.

### 2.5 Schedule-derived exposure descriptors

Learning-stage exposure sequences were reconstructed from the pre-defined learning schedules after excluding formal test stages: staged study and review modules in the structured-learning condition and sequential item arrays in the control condition. Schedule-derived descriptors were then calculated, including cumulative unique-item coverage, the share of exposures devoted to newly introduced items, practice density, and the recurrence lag between repeated exposures, to summarize how the two conditions differed in staged introduction, cumulative review, novelty distribution, and density of exposure opportunities. Because these descriptors were derived from the implemented learning schedules and not independently manipulated, they were interpreted as exploratory contextual measures rather than as causal factors.

### 2.6 Statistical analysis

Correct-trial log-transformed response time during Sessions 1–4 was treated as the principal inferential outcome, with total learning time, response-time variability, and inverse efficiency score analyzed as complementary outcomes. Response times were log-transformed to address positive skew (van der Linden, 2006, 2009, 2019), and conservative participant-level trimming was applied. Sensitivity analyses confirmed robustness across alternative preprocessing specifications, including no trimming, IQR-based trimming, and participant-level median-based summaries.

Trial-level generalized estimating equation (GEE) models examined between-group differences in learning-phase response time while accounting for repeated observations clustered within participants (Liang and Zeger, 1986), using a Gaussian family with an identity link, an exchangeable working correlation structure, and robust standard errors. Additional robustness models were adjusted for item identity and pre-defined stimulus difficulty level. Participant-level models were also fitted for mean log-transformed response time, log-transformed inverse efficiency score, total learning time, and learning-phase accuracy, treating group as the principal predictor and baseline pre-test performance as a covariate.

Exploratory models examined whether the session-level practice density was associated with acquisition-phase performance. These analyses assessed descriptive consistency between exposure structure and efficiency; they were not interpreted as mediation analyses or tests of independent component effects. Inferential emphasis was placed on effect sizes, confidence intervals, and cross-specification consistency rather than on isolated p values. All analyses were conducted in Python 3.12 using pandas, NumPy, SciPy, and statsmodels.

## 3 Results

Between-group comparisons focused on process-sensitive outcomes during the learning phase. The main acquisition-efficiency results are summarized in Table 2.

**Table 2:** Learning-phase process-sensitive outcomes in the structured-learning and control groups.

Outcome	Structured learning	Control	Mean difference (95% CI)	*p* value	Hedges' *g*
Correct-trial RT during learning (ms)	3, 829 ±1, 083	5, 101 ±1, 692	−1, 273 (−1, 988 to −557)	<0.001	−0.88
Trial-level GEE group effect on correct-trial logRT (control vs. structured)	—	—	β = 0.283 (0.146 to 0.420)	<0.001	—
RT variability (SD of correct-trial logRT)	0.670 ±0.115	0.682 ±0.133	−0.012 (−0.075 to 0.051)	0.703	−0.10
Total learning time (min)	52.72 ±6.74	62.53 ±6.73	−9.81 (−13.21 to −6.42)	<0.001	−1.44
Repetition count across learning stages	936.68 ±39.96	885.22 ±67.33	51.46 (23.55 to 79.37)	0.001	0.91
Inverse efficiency score (IES)	3, 712.87 ±1, 506.58	5, 430.81 ±1, 639.99	−1, 717.94 (−2, 510.98 to −924.91)	< 0.001	−1.08

Values are mean ± SD unless otherwise indicated. Group differences are shown as structured learning minus control or as specified in the table. Repetition count is presented as a schedule-derived descriptor of the implemented learning structure rather than as a principal inferential endpoint. RT, response time; logRT, log-transformed response time; GEE, generalized estimating equation.

### 3.1 Learning-phase perceptual decoding time and robustness

**Figure 1:**
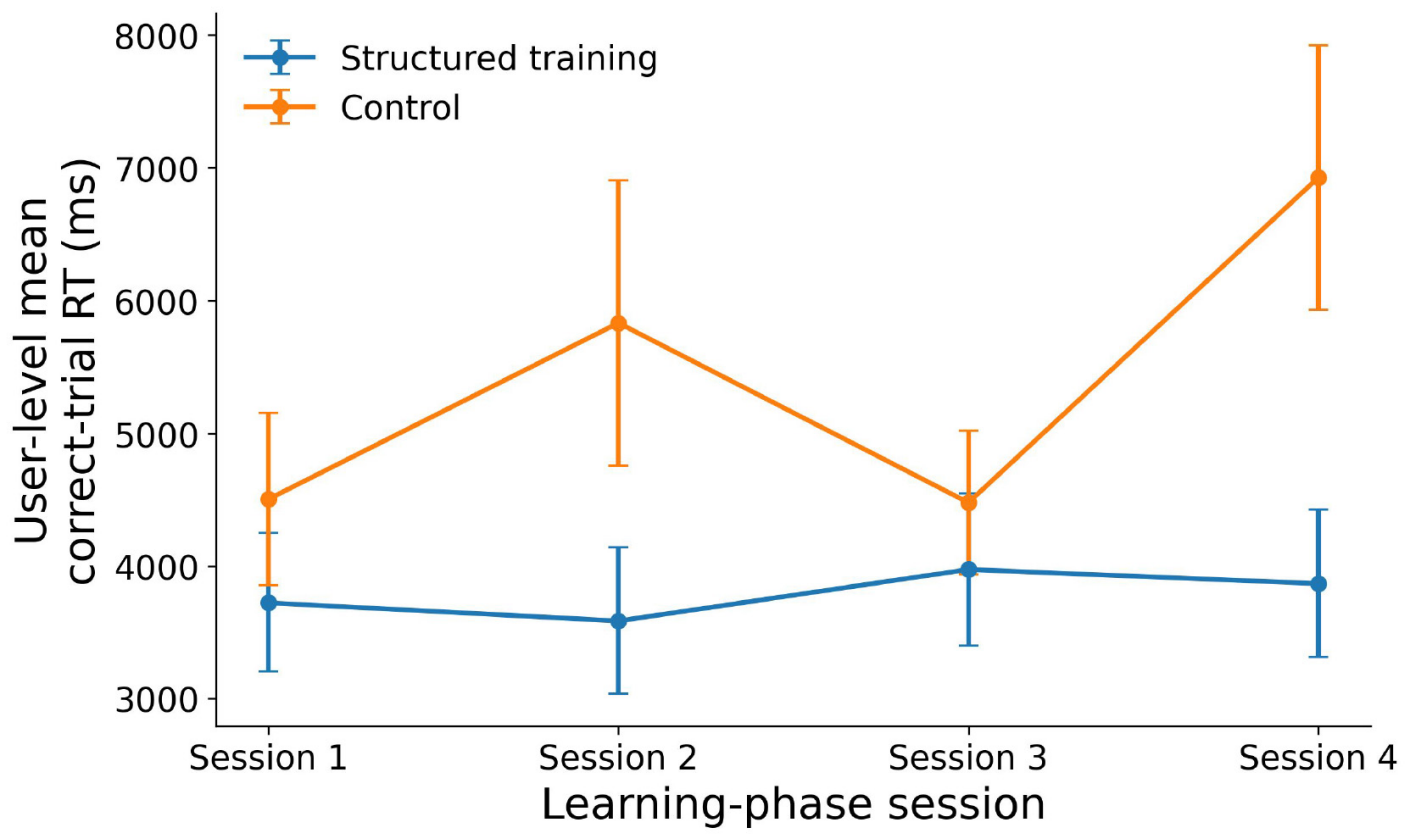
Session-wise trajectory of learning-phase correct-trial response time in the structured-learning and control conditions. Values represent session-wise mean correct-trial response times during the learning phase. Sample sizes were *n* = 31 for the structured-learning group and *n* = 32 for the control group. Error bars indicate standard errors.

Participant-level mean correct-trial response time during Sessions 1–4 was shorter in the structured-learning group than in the control group (3,829 ± 1,083 ms vs. 5,101 ± 1,692 ms; mean difference = –1,273 ms, 95% CI –1,988 to –557; *p* < 0.001; Hedges' *g* = –0.88), corresponding to an approximately 25.0% relative reduction. As shown in Figure 1, the structured-learning group displayed shorter correct-trial response times across the session-wise learning trajectory.

Response-time trimming had minimal impact. Among the 1,899 eligible correct-trial observations across Sessions 1–4, only five observations (0.26%) were excluded. After trimming, analyzable correct-trial counts were 424, 386, 798, and 286 for Sessions 1–4, respectively. Session-specific counts by group were 214 and 210 in Session 1, 195 and 191 in Session 2, 399 and 399 in Session 3, and 157 and 129 in Session 4 for the structured-learning and control groups, respectively.

The primary trial-level GEE model confirmed a significant group effect on the correct-trial log-transformed response time (β = 0.283, 95% CI 0.146 to 0.420; *p* < 0.001), corresponding to an approximately 32.7% increase in the control group on the original scale. A supplementary model including session and session-by-group interaction terms yielded a significant overall interaction (χ^2^ = 28.96, df = 3, *p* < 0.001). Because session-specific test content was not identical across sessions, these interaction results were interpreted as occasion-specific differences rather than as pure longitudinal growth parameters.

The response-time advantage remained robust across alternative preprocessing specifications, including no trimming (–1,194 ms, *p* = 0.0013), IQR-based trimming (–1,242 ms, *p* = 0.0002), and participant-level median-based summaries (–1,189 ms, **p** = 0.0002). The group difference also remained evident after adjustment for item identity (β = 0.333, SE = 0.065, *p* < 0.001) and pre-defined shape-difficulty level (β = 0.324, SE = 0.065, *p* < 0.001).

### 3.2 Temporal acquisition cost, response stability, integrated efficiency, and practice structure

Response-time variability did not differ meaningfully between groups (0.670 ± 0.115 vs. 0.682 ± 0.133; mean difference = –0.012, 95% CI –0.075 to 0.051; *p* = 0.703; Hedges' *g* = -0.10). Thus, shorter response times under structured learning were not accompanied by clear evidence of greater response instability.

**Figure 2:**
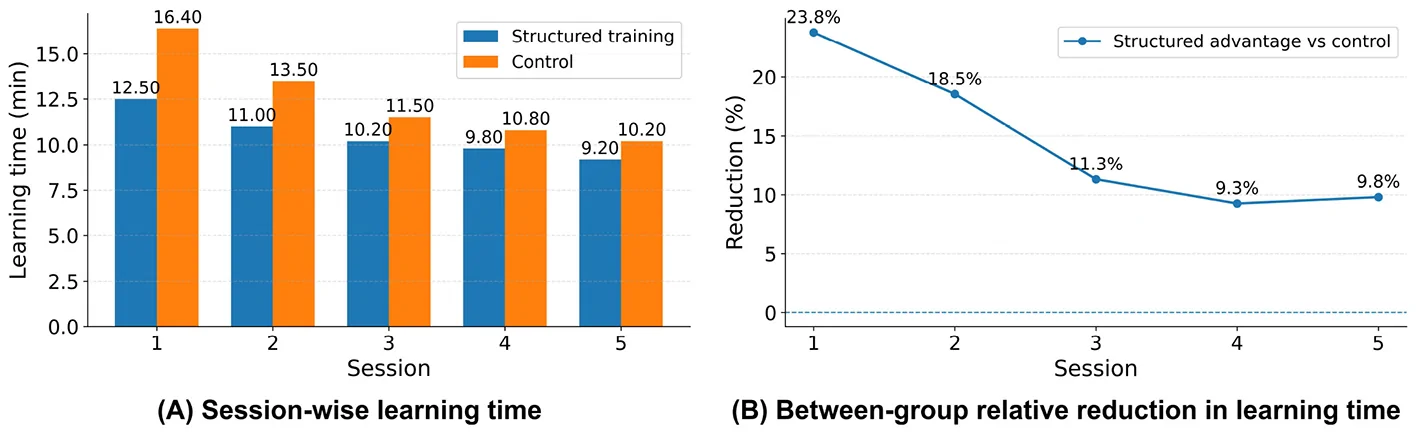
Session-wise learning-stage time and between-group relative reduction across Sessions 1–5. **(A)** Session-wise learning-stage duration by learning condition. Values represent descriptive summaries of learning-stage time only and exclude test-related stages. **(B)** Between-group relative reduction in learning-stage time for the structured-learning condition relative to the control condition, expressed as a percentage at each session. Sample sizes were *n* = 31 for the structured-learning group and *n* = 32 for the control group. This figure provides descriptive context for temporal acquisition cost and should not be interpreted as a confirmatory time-varying analysis; no inferential error bars are shown.

Structured learning was associated with a lower total learning time across the learning stages in Sessions 1–5 (52.72 ± 6.74 min vs. 62.53 ± 6.73 min; mean difference = –9.81 min, 95% CI –13.21 to –6.42; *p* < 0.001; Hedges' *g* = –1.44), corresponding to a 15.7% relative reduction. Figure 2 presents session-resolved summaries of learning-stage time, with larger between-group separation in Sessions 1 and 2 and a narrower difference in later sessions.

The repetition count was higher in the structured-learning group than in the control group (936.68 ± 39.96 vs. 885.22 ± 67.33; mean difference = 51.46, 95% CI 23.55 to 79.37; *p* = 0.001; Hedges' *g* = 0.91). Because repetition exposure was protocol-linked by design, this measure is presented as a schedule-derived descriptor rather than as a principal inferential endpoint. The higher repetition count occurred alongside a shorter total learning time.

**Figure 3:**
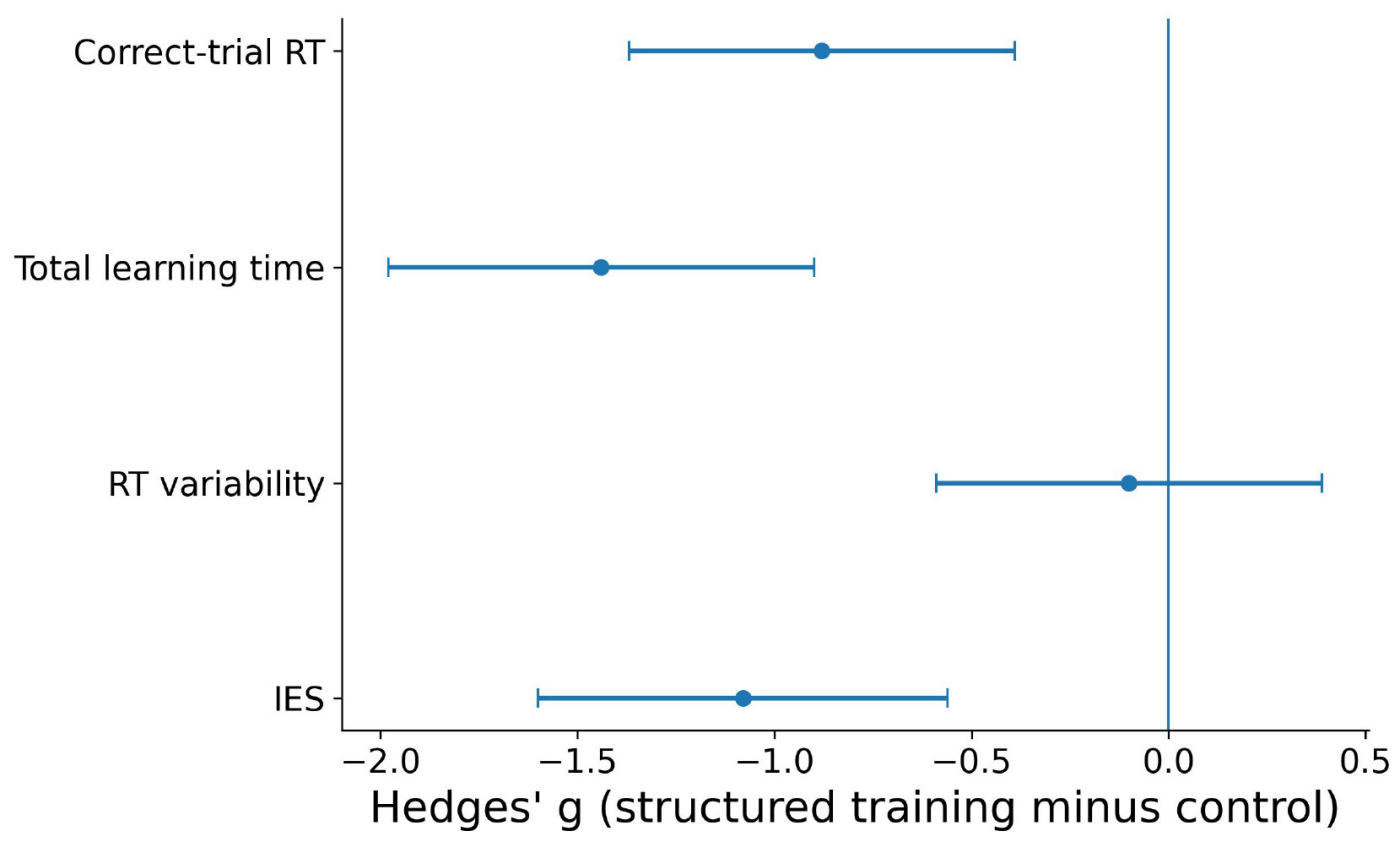
Acquisition-efficiency profile across principal process-sensitive outcomes. Effect sizes are reported as Hedges' g for structured learning minus control. Sample sizes were *n* = 31 for the structured-learning group and *n* = 32 for the control group. Negative values favor structured learning for correct-trial response time, total learning time, response-time variability, and inverse efficiency score. RT, response time; ToT, total learning time; IES, inverse efficiency score.

The inverse efficiency score was lower in the structured-learning group than in the control group (3,713 ± 1,507 vs. 5,431 ± 1,640; mean difference = –1,718, 95% CI –2,511 to -925; *p* < 0.001; Hedges' *g* = –1.08), corresponding to a 31.6% relative reduction. As shown in Figure 3, structured learning showed more favorable values for correct-trial response time, total learning time, and inverse efficiency score, whereas response-time variability showed little between-group separation.

### 3.3 Pre-test-adjusted robustness analyses

Baseline pre-test performance did not differ meaningfully between groups (structured learning: 9.29 ± 3.99 vs. control: 9.84 ± 3.98; *p* = 0.584). In the participant-level models adjusting for pre-test performance, the principal findings remained materially unchanged: the control group continued to show longer log-transformed response time, higher log-transformed inverse efficiency score, and longer total learning time. Learning-phase accuracy did not show an adjusted between-group difference of comparable magnitude. These results indicate that the response-time, total-learning-time, and inverse-efficiency-score differences were not explained by baseline pre-test differences.

### 3.4 Schedule-derived exposure structure and practice density

**Figure 4:**
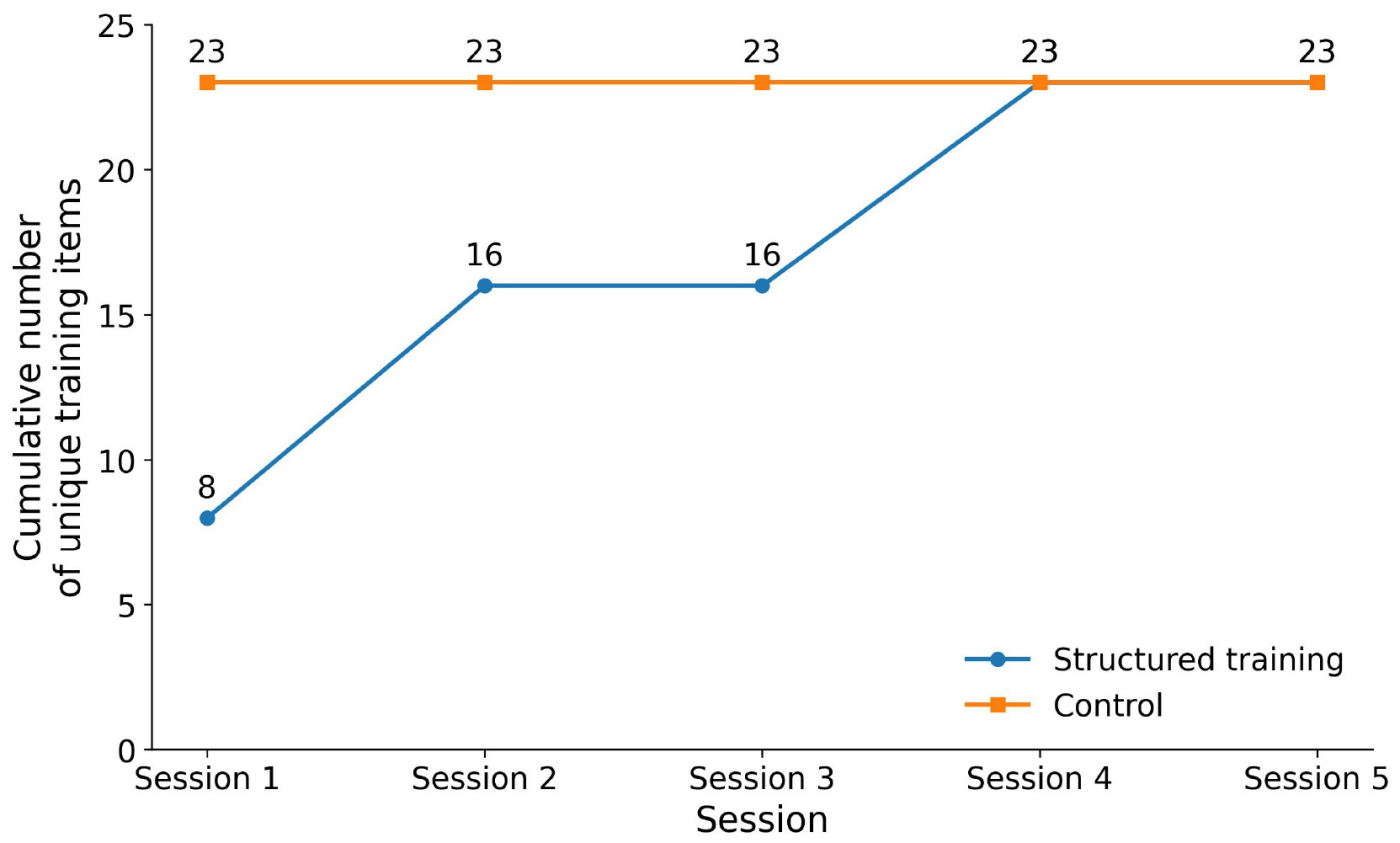
Cumulative unique-item coverage across learning sessions in the structured-learning and control protocols. Values indicate the cumulative number of distinct learning items scripted for exposure by each session after excluding formal test stages. The structured-learning protocol showed staged expansion of item coverage, whereas the control protocol exposed learners to the full item set from Session 1 onward. No participant-level error bars are shown because this figure describes protocol-level item coverage rather than participant-level variation.

Schedule-derived descriptors showed clear differences in item exposure organization across sessions (Figure 4). The structured-learning protocol increased cumulative unique-item coverage in a staged manner from eight items in Session 1 to 16 items by Session 2, retained 16 items in Session 3, and then expanded to the full 23-item set by Session 4. In contrast, the control protocol exposed learners to the full 23-item set from Session 1 onward.

**Figure 5:**
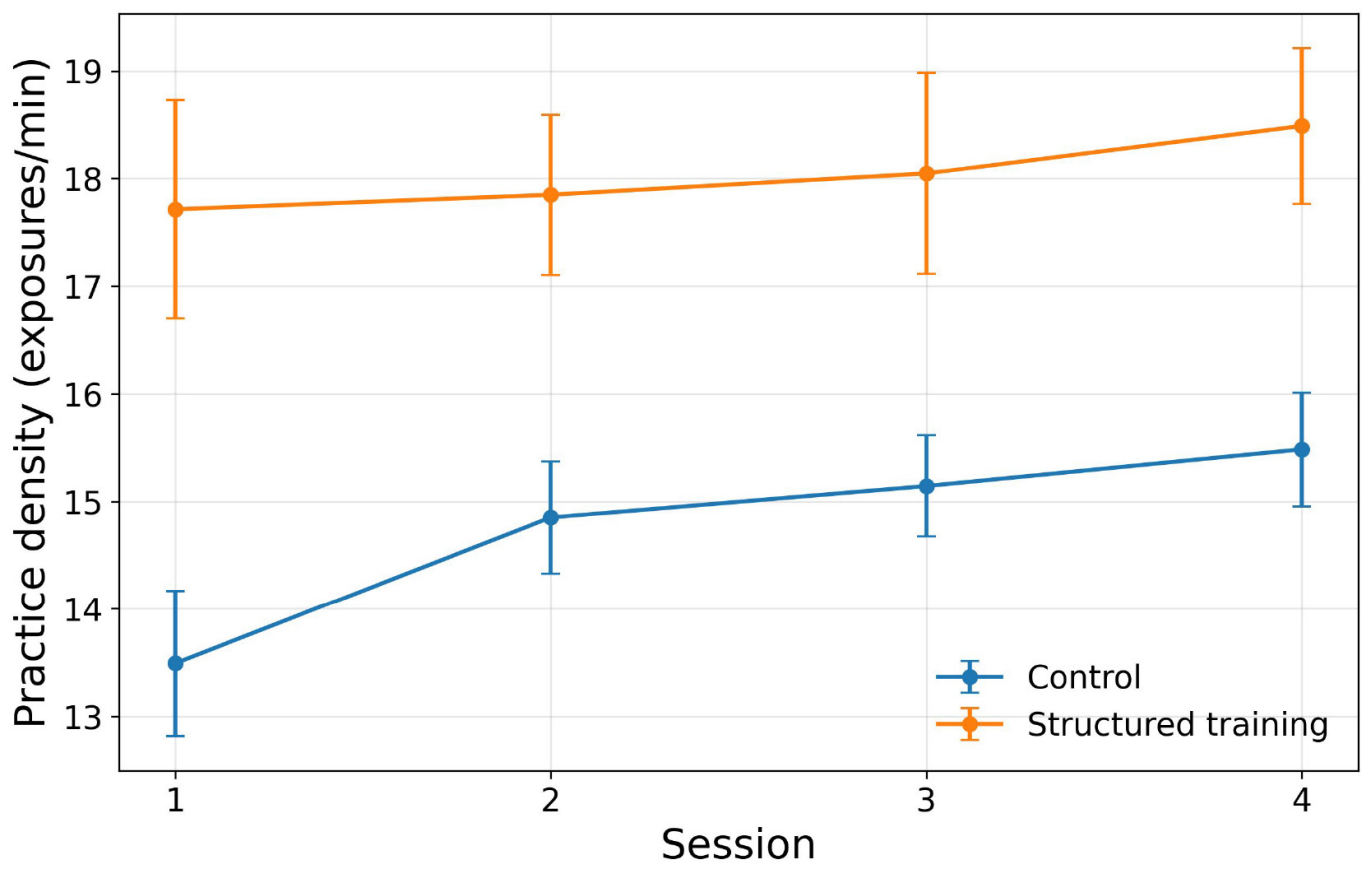
Session-wise practice density during the acquisition phase by learning condition. Acquisition-phase sessions correspond to Sessions 1–4. Sample sizes were *n* = 31 for the structured-learning group and *n* = 32 for the control group. Error bars indicate 95% confidence intervals. Higher values indicate denser effective learning-stage exposure opportunities delivered per minute under the implemented learning-stage exposure structure.

In the structured-learning condition, the new-item exposure share was 100.0% in Session 1, 45.5% in Session 2, 0.0% in Session 3, 42.2% in Session 4, and 0.0% in Session 5; in the control condition, it was 100.0% in Session 1 and 0.0% in all subsequent sessions. During Sessions 1–4, practice density was higher in the structured-learning condition than in the control condition (18.02 vs. 14.74 exposures/min; approximately 22.2% denser), mean learning-stage time per session was lower (10.47 vs. 12.59 min), and mean recurrence lag was shorter (5.11 vs. 22.81 sequence positions; Figure 5). Together, these schedule-derived differences indicate that the structured-learning package differed from the control protocol in item-ordering logic, novelty distribution, review structure, and exposure density.

### 3.5 Exploratory protocol-linked analyses of practice density

Supplementary GEE models incorporated session-level practice density as an exploratory explanatory marker. For correct-trial log-transformed response time, higher practice density was associated with shorter response time (β = –0.057, SE = 0.011, 95% CI –0.079 to –0.036, *p* < 0.001). After practice density was included, the between-group coefficient was attenuated from 0.291 (SE = 0.071, 95% CI 0.151 to 0.430, *p* < 0.001) to 0.105 (SE = 0.082, 95% CI –0.057 to 0.266, *p* = 0.203).

A parallel pattern was observed for log-transformed inverse efficiency score. The group coefficient was reduced from 0.508 (SE = 0.083, 95% CI 0.347 to 0.670, *p* < 0.001) to 0.306 (SE = 0.109, 95% CI 0.093 to 0.519, *p* = 0.005) after inclusion of practice density. Practice density itself remained associated with lower inverse efficiency score (β = –0.060, SE = 0.018, 95% CI –0.095 to –0.026, *p* = 0.001). These supplementary analyses provide protocol-linked explanatory context but should not be interpreted as evidence of mediation or the isolated effect of any single learning component.

### 3.6 Descriptive decomposition of learning-stage time within the structured-learning condition

Within the structured-learning condition, quiz-related activity accounted for the largest share of learning-stage time (60.2%), followed by compare-and-contrast presentation (15.7%), compare-and-contrast selection (15.4%), and shape introduction (8.7%). Review stages showed a higher quiz-time proportion than study stages (65.1 vs. 54.6%), consistent with the intended logic of cumulative review and retrieval-oriented practice.

These descriptive summaries clarify the internal composition of the structured-learning package and should not be interpreted as evidence of independent component contributions.

## 4 Discussion

The present study provides behavioral-log evidence that structured learning was associated with greater early acquisition efficiency in visual-to-auditory sensory substitution. Rather than re-evaluating endpoint recognition achievement, the analysis focused on how efficiently learners acquired usable auditory representations of visual shape information during the learning phase. These findings indicate that early acquisition efficiency in sensory-substitution learning may be related not only to how visual information is transformed into sound, but also to how sensory mappings are introduced, sequenced, and repeatedly practiced during learning. These findings are relevant to perception science because they provide behavioral-log evidence that the temporal and structural organization of sensory exposure is associated with early perceptual decoding efficiency in a controlled sensory-substitution learning model.

The response-time advantage remained evident across analytic specifications and after adjustment for baseline performance, supporting the interpretation that the observed pattern reflected a robust acquisition-phase efficiency difference rather than a preprocessing or model-specific artifact. At the same time, learning-phase accuracy did not show a between-group difference of comparable magnitude after baseline adjustment. Together with the inverse efficiency score findings, these results are most consistent with an efficiency-related acquisition advantage rather than with a generalized superiority claim about overall achievement. In other words, structured learning was associated with more efficient early perceptual decoding without clear evidence of a detrimental speed-accuracy trade-off.

The schedule-derived analyses provide package-level explanatory context for the observed acquisition-efficiency pattern. Relative to the control condition, the structured-learning package distributed item novelty across sessions, interleaved new-item introduction with cumulative review, and delivered denser scheduled exposure opportunities within less total learning-stage time. These characteristics are descriptively compatible with lower concurrent novelty demands and more efficient repeated exposure within the available learning window. This interpretation is consistent with broader perceptual-learning and cognitive-psychology perspectives suggesting that structured organization of foundational features and staged grouping of stimuli can reduce the processing demands of early learning, while repeated exposure and cumulative review may support stabilization of emerging perceptual mappings (Cepeda et al., 2008; Paas and van Merriënboer, 1993; Schmidt and Bjork, 1992; Soderstrom and Bjork, 2015; Sweller et al., 2011; Winstein and Schmidt, 1990). However, because practice density and related schedule-derived variables were not independently manipulated, the present data do not support formal mediation claims or causal attribution to any single learning component. Consequently, the findings are best interpreted at the level of the structured-learning package as an integrated whole, not as evidence for the independent effect of any single component.

From a perceptual-learning perspective, basic-shape learning is better understood not as a trivial endpoint, but as a controlled substrate for examining how learners acquire foundational image-to-sound mappings. This framing is consistent with studies on perceptual learning and object recognition emphasizing the acquisition and organization of discriminative features (Biederman, 1987; Gibson, 1969; Sweller et al., 2011). Prior sensory-substitution studies further suggest that learning basic sound-visual mappings can support recognition and may provide a basis for subsequent generalization to more complex stimuli and functional use (Auvray et al., 2007; Kucinkas et al., 2026; Poirier et al., 2007; Reich and Amedi, 2015). Although such downstream transfer was not directly tested in the present study, the current findings suggest that the organization of early learning may be related to how efficiently learners enter successful sound-visual mapping acquisition. The present findings should be understood as complementary to, rather than duplicative of, the prior achievement-focused report; the two manuscripts contribute different inferential information from the same experimental platform.

The broader implication is that sensory-substitution research should evaluate acquisition efficiency alongside endpoint recognition accuracy. A sensory code may be learnable in principle, but its perceptual usability also depends on how efficiently learners can enter a stable decoding process. The magnitude of the observed reductions (approximately 25% in correct-trial response time, 16% in total learning time, and 32% in inverse efficiency score) suggests that structured learning may meaningfully reduce the temporal burden of early sensory-substitution acquisition without compromising accuracy. In practical training contexts, faster and more efficient early decoding may help reduce cognitive load and fatigue, support sustained engagement, and increase the feasibility of repeated practice before more complex or ecologically valid sensory-substitution tasks are introduced. Together with prior work showing that self-training, mapping flexibility, exploration strategy, self-motion, and task context can influence sensory-substitution performance (Akar et al., 2025; Bordeau et al., 2025; Buchs et al., 2021; Kucinkas et al., 2026; Stiles et al., 2015), the present findings suggest that the organization of early learning should be treated as part of the perceptual-learning problem rather than as a neutral procedural detail. Structured learning should therefore be considered as a relevant design dimension for early cross-modal perceptual acquisition. These practical implications should, however, be interpreted cautiously because the present findings are based on blindfolded sighted adults rather than end-users with visual impairments. The study therefore does not establish whether the observed acquisition-efficiency advantage directly generalizes to clinical or applied settings, downstream functional application, or real-world assistive benefit for visually impaired users. Future work should test whether similar learning-efficiency advantages generalize to end-user populations, longer training periods, more complex stimulus classes, ecologically relevant sensory-substitution tasks, and complementary psychophysical measures of perceptual learning.

A related boundary of interpretation concerns the role of the fixed sensory-conversion framework: the analysis held this framework constant and examined whether learning structure was associated with acquisition-phase efficiency, including response speed, total learning time, response-time stability, and integrated speed–accuracy performance. This distinction is important in light of evidence that acoustic frequency properties can influence cross-modal awareness, attention, and readiness to respond. Previous studies have shown that sounds can enhance awareness of visual events through attentional mechanisms (Pápai and Soto-Faraco, 2017), that combining visual contrast information with frequency-defined sound properties can facilitate faster decisions (Dresp-Langley and Monfouga, 2019), and that frequency-specific stimulation can modulate cognitive, emotional, and sensory processing in ways relevant to learning and rehabilitation, suggesting potential relevance for frequency-controlled sound approaches in cognitive, emotional, and motor recovery in clinical settings (Yang et al., 2025). These findings suggest that frequency-selective auditory properties may be an important component of sensory-substitution design. However, the present study did not manipulate acoustic frequency ranges or frequency–feature assignments and was not designed to isolate frequency-specific effects. The choice of a fixed conversion framework was made to hold the encoding dimension constant so that the contribution of learning structure to acquisition efficiency could be examined under controlled encoding conditions. Future studies should therefore examine how frequency-controlled mapping designs interact with structured learning methods to jointly optimize V2A-SS acquisition and usability.

## 5 Limitations

Several limitations define the claim boundary of the present study. First, the study relied on behavioral acquisition-phase measures and should therefore be interpreted as evidence of acquisition-efficiency patterns in perceptual learning rather than as direct evidence of subjective perceptual experience, internal perceptual mechanisms, or real-world assistive performance.

Second, the present sample consisted of blindfolded sighted adults rather than people who are blind or have low vision. This approach is commonly used in sensory-substitution research as a controlled behavioral testbed for examining early learning and pattern recognition with substituted sensory mappings under reduced heterogeneity (Auvray et al., 2007; Jicol et al., 2020; Poirier et al., 2007; Proulx et al., 2014). In the present study, it helped minimize variability related to residual vision, prior device-use experience, and task familiarity, thereby enabling a clearer examination of early acquisition efficiency under comparable sensory-input conditions. Nevertheless, people who are blind or have low vision may differ from blindfolded sighted adults in ways that could alter the magnitude or pattern of learning-structure effects observed in the present study. Long-term visual deprivation is associated with experience-dependent cross-modal plasticity, including the recruitment of visual cortical regions for auditory and tactile processing (Amedi et al., 2007; Striem-Amit et al., 2012a,b). Such neuroplastic differences may influence the rate at which auditory representations of visual structure are acquired, the stability of those representations across trials, and the extent to which staged item introduction or cumulative review supports early perceptual decoding. At the same time, prior V2A-SS studies have reported no statistically significant recognition-performance differences between blind participants and blindfolded sighted participants under specific task conditions (Abboud et al., 2014; Striem-Amit et al., 2012b). Such null-performance-difference findings suggest that blindfolded sighted samples may provide useful controlled evidence for certain aspects of sensory-substitution learning and pattern recognition. However, these findings do not eliminate the need to test whether the present acquisition-efficiency pattern generalizes to actual users with visual impairments. Future studies should therefore replicate the acquisition-phase analysis with visually impaired participants to determine whether similar efficiency advantages generalize across populations with different sensory histories, neuroplastic profiles, and compensatory strategies.

Third, the learning task focused on basic shape categories as a controlled perceptual-learning substrate. This stimulus set was appropriate for isolating foundational sound-visual mapping acquisition, but it does not establish whether the observed efficiency advantages would extend to more complex and ecologically valid visual environments, such as natural scenes, dynamic objects, or navigation-relevant stimuli.

Fourth, the study evaluated the structured-learning protocol as an integrated package and did not establish a causal mechanism at the level of isolated learning components. Specifically, staged item introduction, cumulative review, compare-and-contrast scaffolding, and the resulting differences in scheduled practice density were combined within this package; therefore, the present data cannot determine the independent contribution of any single instructional component. Future studies employing factorial or component-isolation designs are needed to dissociate the relative contributions of each element to acquisition efficiency.

Finally, because the analyzed outcomes were not prospectively specified as primary endpoints at the time of experimental implementation, the findings are best interpreted in terms of convergent patterning across related efficiency-sensitive behavioral indicators rather than as a definitive confirmatory test of a pre-specified mechanism. This limitation does not negate the consistency observed across the efficiency-related outcomes; rather, it requires that the findings be interpreted as behavioral evidence from an acquisition-phase analysis rather than as a prospective mechanistic test.

## 6 Conclusions

In this controlled visual-to-auditory sensory-substitution learning model, structured learning was associated with a more efficient early acquisition profile, including shorter correct-trial response time, lower total learning time, and improved integrated speed-accuracy efficiency, without clear evidence of increased response-time instability. These behavioral-log findings suggest that the efficiency of early sound-visual mapping acquisition may be related not only to how visual information is transformed into sound, but also to how those mappings are sequenced and repeatedly practiced during learning. By characterizing acquisition efficiency with process-sensitive behavioral indicators, the study provides evidence on early sound-visual mapping acquisition beyond endpoint achievement alone. The results should therefore be interpreted as evidence from a controlled perceptual-learning model rather than as direct evidence of downstream functional application or real-world assistive benefit. Future studies should examine whether similar acquisition-efficiency advantages extend to end-user populations, longer training periods, and more ecologically valid sensory-substitution tasks.

## Data Availability

The data analyzed in this study is subject to the following licenses/restrictions: owing to institutional and ethical restrictions related to de-identified but linkable human-participant behavioral log data, the raw linked dataset cannot be made publicly available. De-identified derived data and analysis code supporting the conclusions of this article are available from the corresponding author on reasonable request, subject to institutional approval and applicable ethics requirements. Requests to access these datasets should be directed to Kyeong Deok Moon, kdmoon@etri.re.kr.
