# Supplementary material for: Early acquisition efficiency in visual-to-auditory sensory substitution: the role of structured learning
Source: Front Psychol. 2026 Aug 26;17:1887888. doi: 10.3389/fpsyg.2026.1887888 (PMC13553928; doi:10.3389/fpsyg.2026.1887888)
Supplement: Supplementary file 1 [file Data_Sheet_1.pdf]

Supplementary Material

1 SUPPLEMENTARY TABLE S1

Table S1. Comparison of recent V2A-SS studies with respect to outcome focus and acquisition-phase process measurement.

Study	Participants	Primary focus	Main outcomes	Acquisition-phase process metrics?	Distinction from present study
Buchs et al. 2021	Sighted (blindfolded)	Self-training feasibility	Recognition accuracy	No	Endpoint-oriented outcome focus; no integrated acquisition-efficiency profile
Jicol et al. 2020	Sighted and VI	Navigation + self-motion	Localization accuracy	No	Endpoint-oriented outcome focus; no acquisition-phase process analysis
Pasqualotto and Esenkaya 2016	Sighted (blindfolded)	Spatial updating	Accuracy	No	Endpoint-oriented outcome focus; no process-level behavioral logs
Bordeau et al. 2025	Sighted (blindfolded)	Localization by scene layout	Localization ability	Partial (decision time only)	RT only; no ToT, IES, or RT variability
Kucinkas et al. 2026	Sighted and VI	Mapping flexibility and transfer	Accuracy, generalization	No	Endpoint-oriented outcome focus; no acquisition-phase process analysis
Akar et al. 2025	Sighted (blindfolded)	Exploration strategy	Accuracy, strategy types	No	Endpoint-oriented outcome focus; no learning structure manipulation
Moon et al. 2025 (prior report)	Sighted (blindfolded)	Assessment-point achievement	Recognition accuracy at test points	No	Same protocol; present study adds RT, ToT, RT variability, IES, and practice density
Present study	Sighted (blindfolded)	Acquisition-phase efficiency	Correct-trial RT, ToT, RT variability, IES, practice density	Yes—multiple process-sensitive indicators	Extends prior V2A-SS work by jointly analyzing RT, ToT, RT variability, IES, and practice density during acquisition

Note. VI = visually impaired; RT = response time; ToT = total learning time; IES = inverse efficiency score.

2 SUPPLEMENTARY FIGURE S1

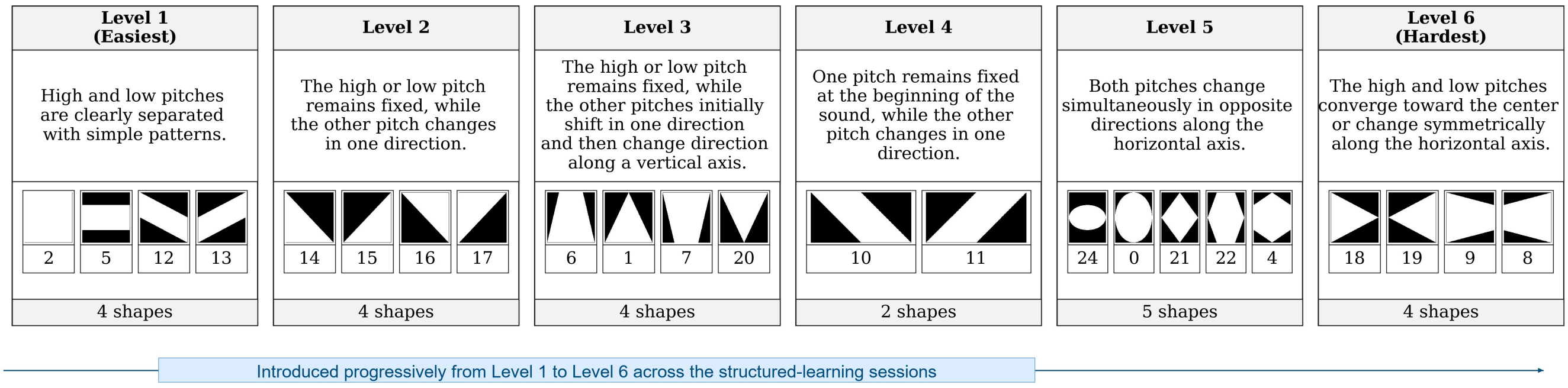

Figure S1. The 23 shape categories organized by auditory-pattern difficulty level (Levels 1–6). Each panel shows the visual shape category, its shape ID, and its difficulty classification. The arrow indicates the progressive introduction of item levels across the structured-learning sessions.

3 SUPPLEMENTARY FIGURE S2

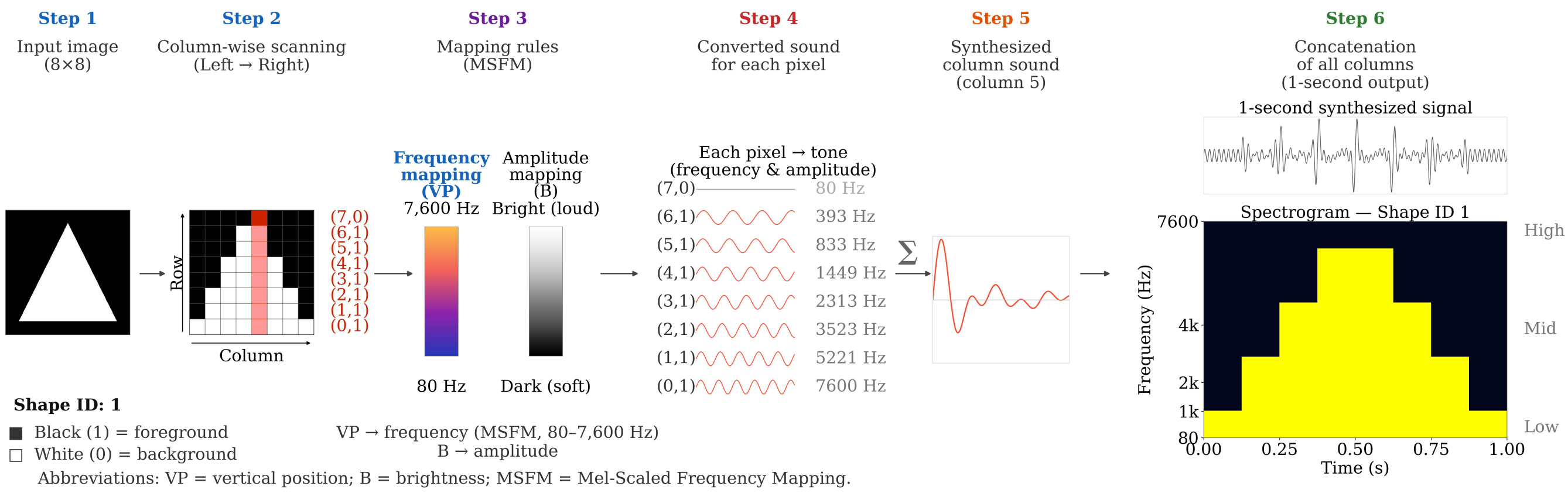

Figure S2. Schematic illustration of the fixed MSFM-optimized visual-to-auditory conversion process. A representative 8×8 shape image (Shape ID 1, isosceles triangle, Level 3) is scanned column by column from left to right. Within each column, vertical pixel position is mapped to frequency and pixel brightness is mapped to amplitude. Pixel-level tones are synthesized into a column sound, and column sounds are concatenated to generate the final one-second auditory output. The schematic summarizes the conversion framework described in the prior report and retained in the present analysis. Abbreviations: VP = vertical position; B = brightness; MSFM = Mel-Scaled Frequency Mapping.
